# A Striking Comparison

**Published:** 1907-04

**Authors:** 


### A Striking Comparison.

"Are Texas Hogs Worth More Than Texas Children?" is a question strikingly set forth in a cartoon which accompanies Walter B. Whitman's article on food adulteration in Texas, which appears in *Holland's Magazine* for November. The cartoon shows a couple of porkers contentedly munching their feed—bearing a State inspection tag—while two children with pinched faces, are eating their morning meal, a bottle of milk labeled formaldehyde conspicuously displayed on the table. The picture brings out freely the fact that while the State of Texas has a feed law which assures the purity of feed sold for live stock and provides for its proper inspection, it makes no provision for the protection of the babies against food adulterations. A dealer who sells impure hog feed subjects himself to heavy penalties, but he may sell food containing poisonous adulterants for human consumption without fear of arrest. *Holland's Magazine* is making a strong campaign for the passage of a pure food law by the next Legislature, and it publishes in its November issue a number of letters from Texas State Senators and Representatives endorsing its work along this line.

The November number is elaborately illustrated and contains a large number of special articles and short stories which place it well alongside of the leading periodicals which come from the Eastern States.

